# Atrial Fibrillation in Patients with Very High Risk for Stroke and Adverse Events—Insights from the Observational ARENA Study

**DOI:** 10.3390/jcm13226645

**Published:** 2024-11-06

**Authors:** Angelika Alonso, Ibrahim Akin, Matthias Hochadel, Martin Borggrefe, Hendrik Lesch, Armin Grau, Ralf Zahn, Patrick Lugenbiel, Christopher Jan Schwarzbach, Tim Sueselbeck, Jochen Senges, Christian Fastner

**Affiliations:** 1 Department of Neurology, Mannheim Center for Translational Neuroscience, University Hospital Mannheim of University of Heidelberg, Faculty of Medicine Mannheim, Universitätsklinikum Mannheim, 68167 Mannheim, Germany; hendrik.lesch@umm.de; 2 Department of Cardiology, Haemostaseology and Medical Intensive Care, University Medical Center Mannheim (UMM), Medical Faculty Mannheim, Heidelberg University, European Center for AngioScience (ECAS) and German Center for Cardiovascular Research (DZHK) Partner Site Heidelberg/Mannheim, 68167 Mannheim, Germany; ibrahim.akin@umm.de (I.A.); martin.borggrefe@umm.de (M.B.); christian.fastner@umm.de (C.F.); 3 Stiftung Institut für Herzinfarktforschung (Stiftung IHF), 67063 Ludwigshafen, Germany; hochadel@ihf.de (M.H.); senges@stiftung-ihf.de (J.S.); 4 Department of Neurology, Klinikum der Stadt Ludwigshafen am Rhein gGmbH, 67063 Ludwigshafen, Germanyschwarch@klilu.de (C.J.S.); 5 Department of Cardiology, Pneumology, Angiology and Medical Intensive Care, Klinikum der Stadt Ludwigshafen am Rhein gGmbH, 67063 Ludwigshafen, Germany; zahnr@klilu.de; 6 Department of Cardiology, Angiology and Pneumology, Heidelberg University Hospital, 69120 Heidelberg, Germany; patrick.lugenbiel@med.uni-heidelberg.de; 7 Outpatient Clinic for Cardiology, 67071 Ludwigshafen, Germany

**Keywords:** atrial fibrillation, stroke, cerebrovascular event, echocardiography, mortality

## Abstract

**Background:** Atrial fibrillation (AF) is a major cause of stroke. An individual risk estimation remains challenging, as AF patients with and without cerebrovascular event (CVE) may differ in yet unknown factors beyond those covered by the CHA_2_DS_2_-VASc score. We aimed to identify differences between AF patients with and without CVE with regard to AF characteristics and treatment, vascular risk factors and comorbidities, prognosis and outcome. **Methods:** We analyzed patients included in the Atrial Fibrillation Rhine-Neckar Region (ARENA) Project, an observational cohort study of patients with AF. Patients were recruited by their general practitioner or during a hospital stay and were divided into two groups for the present analysis: patients with acute CVE at baseline and/or history of CVE versus patients without CVE. Follow-up at 1 year was conducted via phone call. **Results:** Of 2061 included patients (60.6% male), 292 (14.2%) belonged to the CVE group. Patients in the CVE group were older (mean age 74.6 versus 71.7 years; *p* < 0.001) and had a higher CHA_2_DS_2_-VASc score at baseline (5.3 versus 3.3 points; *p* < 0.001) based on the preceding CVE. Moreover, patients with either acute or chronic CVE had a larger left atrium (median diameter 47/46 mm versus 44 mm; *p* = 0.001). Patients with acute CVE had structural heart diseases (*p* < 0.001) less frequently than patients with previous or without CVE. Mortality at 1 year (HR 1.95; 95%-CI 1.37–2.78) was more frequent in the CVE group (*p* < 0.001). During 1-year of follow-up, stroke occurred more frequently in survivors with CVE (2.9% versus 0%; *p* < 0.001). **Conclusions:** AF patients with CVE have a significantly worse prognosis than AF patients without CVE. Atrial structural remodeling, underlying cardiovascular disease, stroke-induced heart injury and further unidentified factors may account for this finding. Characterization of AF patients including echocardiography to detect atrial structural remodeling may be helpful in risk stratification beyond classical scores.

## 1. Introduction

Atrial fibrillation (AF) is the most common arrhythmia in adults, with an estimated prevalence of 2–4% in the general population and a steep increase with age [1]. AF is an independent risk factor for stroke [2], and cardiac embolism accounts for about 20–30% of all strokes [3]. In addition, AF could be detected by implantable devices in cryptogenic stroke patients in up to 30% over a screening period of 3 years [4]. In turn, the prevalence of silent brain infarcts possibly related to cardioembolism in AF patients is about 15% [5], and AF patients with silent brain infarcts have an increased risk of stroke recurrence [6]. The risk of cardioembolic stroke varies in dependence on specific patient characteristics and modifiers that are reflected in the CHA_2_DS_2_-VASc score [7]. The included parameters congestive heart failure, hypertension, age, diabetes mellitus and vascular disease increase the risk of stroke independently of the presence of AF, but in turn are associated with a higher risk for incident AF [1]. Female sex serves as a modifier towards a stronger weighting of the combination with other risk factors [8]. The sensitivity to detect patients with low stroke risk is good [9]. However, while the CHA_2_DS_2_-VASc score can reliably predict patients at low risk for thromboembolic complications, it performs only modestly in discriminating patients with very high risk of embolic stroke (ceiling effect) [7]. Consequently, factors or mechanisms beyond the mere diagnosis of AF and presence of vascular risk factors that modify the stroke risk in AF patients have to be assumed. These might comprise clinical conditions such as chronic kidney disease, but also echocardiographic, biochemical or coagulation parameters and notably yet unknown factors [10]. In our observational cohort study of AF patients with and without cerebrovascular event (CVE), we aimed to identify differences in terms of AF characteristics and treatment, vascular risk factors and comorbidities as well as prognosis and outcome that may help to further differentiate AF patients at very high risk of stroke.

## 2. Materials and Methods

The Atrial Fibrillation Rhine-Neckar Region (ARENA) Project is an observational study of patients with AF in the polycentric Rhine-Neckar Metropolitan Region in Germany with more than 2 million inhabitants. The study was conducted by the Foundation Institut für Herzinfarktforschung (IHF) in cooperation with the Departments of Cardiology of the Medical Faculties Mannheim and Heidelberg, Heidelberg University, the Departments of Neurology of the Medical Faculty Mannheim, Heidelberg University, and the Hospital Ludwigshafen, as well as with local resident cardiologists and the Department of Clinical Pharmacology and Pharmacological Epidemiology, Heidelberg University, in Germany. The aim of the project is an improvement of patient-centered care and prognosis in patients with AF, with a focus on the improvement of stroke prophylaxis [11]. The study was approved by the Ethics Committee of the Rhineland-Palatine State Medical Association (#837.366.15), the Ethics Committee of the Medical Faculty, Heidelberg University (#B-F-2016-051), and the Ethics Committee II of the Medical Faculty Mannheim, Heidelberg University (#2016-613N-MA).

In the present analysis, patients recruited by their resident cardiologists or during a hospital stay were included. To reduce selection bias, consecutive recruitment of all patients fulfilling the inclusion criteria was targeted. Inclusion criteria were (1) adult patient with place of residence in the Rhine-Neckar Metropolitan Region, (2) firstly diagnosed or known AF, (3) written informed consent. The current study is based on the predefined substudy “ARENA intervention” that aimed to examine the effectiveness of population education. For the present analysis, AF patients were grouped according to CVE versus non-CVE. Following the hypothesis that CVE is a distinguishing feature of a very high risk population [6], patients with both acute and/or previous CVE were included in the CVE group. To account for potential differences between patients with acute versus previous CVE, a subanalysis differentiating these patient groups was performed. The following parameters were recorded: baseline characteristics, CVE type, vascular risk factors, cardiac and other relevant comorbidities, technical results (e.g., transthoracic echocardiography, TTE), AF characteristics (e.g., risk scores (CHA_2_DS_2_-VASc score, HAS-BLED score), AF-related symptoms (based on European Heart Rhythm Association, EHRA, classification)), as well as medical and non-medical treatment of AF at baseline. At 365 days, a central follow-up of all patients was scheduled via postal questionnaire or phone call. In detail, current medication and quality of life (EQ-5D) were assessed by postal questionnaires, whereas adverse events and AF symptom burden were assessed by phone call. In case of severe adverse events such as stroke, peripheral embolism or major bleeding, medical reports from hospital admissions were requested. Inquiries were made with registration offices if patients could not be contacted by phone call. Mortality, cause of death, non-fatal adverse events among survivors, such as myocardial infarction (MI), CVE, major bleeding complications (i.e., bleeding with hemodynamic instability, intracranial bleeding, retroperitoneal bleeding), as well as medical and non-medical AF treatment were recorded.

### Statistics

Statistical analyses were performed with SAS^®^ version 9.4 (SAS Institute, Cary, NC, USA). Continuous data are given as means and standard deviation or as medians and interquartile ranges (25th and 75th percentiles), categorical data as frequencies with group-related percentages. Group comparisons of categorial variables were performed with Pearson Chi^2^ test, and Mann–Whitney–Wilcoxon test was used for ordinal and metric variables. Mortality at 365 days and the incidence of its composites with MI (MACE), with MI or stroke (MACCE) and the combined event of MACCE and major bleeding were evaluated by methods of survival analysis (Kaplan–Meier curves, log-rank test, Cox regression). The non-CVE group was used as reference for the calculation of odds ratios and hazard ratios with 95% confidence intervals, respectively.

## 3. Results

In our analysis, 2061 patients (60.6% male, mean age 72.1 years) were included between August 2016 and December 2018 (Table 1). Of these, 292 (14.2%) had either an acute CVE (stroke or transient ischemic attack, TIA) at the time of inclusion (58/292) or a history of stroke or TIA (234/292). About three quarters of patients (77.1% in the CVE group and 72.7% in the non-CVE group) were included during a hospital stay, while 22.9% and 27.3%, respectively, were included during an outpatient contact. Patients in the CVE group were significantly older and had significantly higher CHA_2_DS_2_-VASc and HAS-BLED scores at baseline (Table 2). The difference in CHA_2_DS_2_-VASc score was mainly driven by the history of CVE, whereas there was no significant difference in the presence of the items congestive heart failure, hypertension, diabetes mellitus, previous myocardial infarction or female sex (Table 1).

In order to analyze differences between patients with acute versus previous CVE, we conducted a subanalysis evaluating cardiovascular comorbidities, echocardiographic findings, and AF characteristics (Table 3). Structural heart disease in total (odds ratio 1.56 (1.07–2.28)), coronary artery disease and peripheral arterial disease (odds ratio 2.87 (1.86–4.43)) were more common in patients with previous CVE, while at the time point of acute CVE, less than half of the patients had known structural heart disease. In patients with previous CVE, the diagnosis of AF was significantly more frequently known at baseline (91.5% versus 83.7%, *p* = 0.002) than in non-CVE patients, and significantly more patients were on treatment with oral anticoagulation (OAC) at inclusion. In patients with acute CVE, paroxysmal AF was significantly more frequent than in patients with previous or without CVE. These patients had a significantly lower AF-related symptom burden, as reflected by the EHRA classification, and were more often asymptomatic.

In more than 80% of patients a TTE was performed. While left ventricular ejection fraction was preserved in the majority of patients, patients with CVE had a significantly larger left atrium (LA) as reflected by the median diameter, and a higher proportion of patients had an enlarged LA diameter > 45 mm. However, in the subgroup of patients with known LA diameter (n = 131 with CVE and n = 797 without CVE), occurrence of CVE was not significantly associated with the timespan following AF diagnosis (median 5 years in patients with CVE versus median 4 years in patients without CVE, *p* = 0.16).

**Figure 1:**
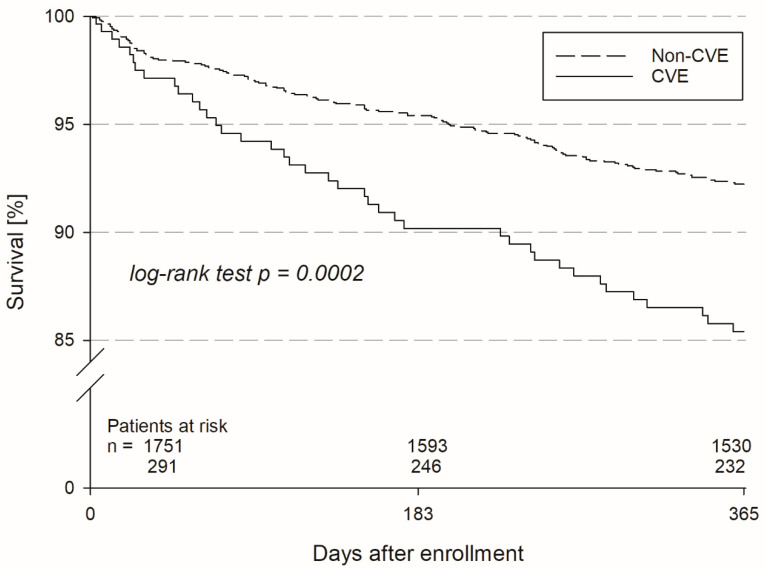
Kaplan–Meier curve, survival analysis for patients without cerebrovascular event (non-CVE) and patients with acute or previous CVE over the follow-up period of 1 year. Of 1752 patients in the non-CVE group, 221 (12.6%) died within 1 year, whereas 59 patients (20.3%) with acute or previous CVE did not survive the follow-up period (*p* = 0.0002).

Follow-up data on vital status were available for 260/292 (89.0%) patients in the CVE group and for 1625/1769 (91.9%) patients in the non-CVE group. One-year mortality was almost twice as high in the CVE group (14.6% versus 7.8%, Figure 1). In line with this, MACE, MACCE and a composite of MACCE and major bleeding were significantly more frequent in the CVE group irrespective of CVE classification as acute or previous CVE (Table 2). Detailed information about adverse events as well as medical and non-medical AF treatment was available for more than half of the patients (for details, see Table 4). The most common reasons for incomplete follow-up or follow-up failure in surviving patients were withdrawal of consent (37.0%), information only via inquiry with registration office (42.1%), and indirect information via surrogates (6.0%), each without significant differences between the CVE and the non-CVE group. Among survivors, stroke occurred significantly more often in the CVE group. Although the percentage of patients taking OAC had increased in both groups, CVE patients were still significantly more frequently treated with OAC in line with a significantly higher CHA_2_DS_2_-VASc score based on the preceding CVE. Left atrial appendage occluders were used in only a few patients in both groups. Concerning non-medical AF treatment, catheter ablation had been performed significantly more often in the non-CVE group.

## 4. Discussion

In the present observational study conducted in a metropolitan region known for its diversity and multiethnicity, AF patients with acute or previous CVE were at significantly higher risk for recurrent stroke, MACE, MACCE or all-cause death within 1 year than AF patients without CVE.

Stroke is the most common and most severe complication in patients with AF [12]. The classical hypothesis proceeds on the assumption of an increased stasis of blood in the LA due to insufficient contraction during AF episodes with rapid and undirected atrial activation, leading to a prothrombogenic activation and clot formation within the LA [13]. However, the temporal association of AF episodes and stroke is weak, which calls such monocausal theory into question: In the The Asymptomatic Atrial Fibrillation and Stroke Evaluation in Pacemaker Patients and the Atrial Fibrillation Reduction Atrial Pacing Trial, only 4/51 pacemaker patients who experienced an acute cardioembolic event had an AF episode within 1 month before stroke or systemic embolism, and only one of these four patients had AF detected at the time of stroke [14]. Recent models emphasize the more comprehensive concept of atrial cardiomyopathy (ACM), integrating not only electrical (i.e., excessive atrial ectopy or AF) but also structural alterations [15,16]. Endothelial dysfunction, inflammation and other factors contribute to fibrosis of the atrial myocardium and LA enlargement, resulting in a prothrombogenic state [16].

Although the true presence ACM was not evaluated in the present study, we could demonstrate that patients in the CVE group had significantly larger LA diameters and a significantly higher proportion with clinically relevantly enlarged LA diameter > 45 mm than non-CVE patients. Even independently of rhythm abnormalities, larger LA dimensions were found to be associated with risk of stroke, as shown in a systematic review of stroke risk in patients in sinus rhythm [17]. Interestingly, structural remodeling with enlargement of LA diameter does not seem to be a pure function of time: whereas LA diameter was associated with occurrence of CVE in our study, duration of AF was not. Consequently, development of LA enlargement is possibly determined by yet unidentified factors. In this context, it is striking that patients with a previous CVE appeared to be treated differently compared to the groups without or with only currently diagnosed CVE: They were less often treated with cardioversion or catheter ablation. Persistent as well as permanent AF is associated with an increased prevalence of heart failure [18]. Alternatively, the cardiovascular multimorbidity of patients with a history of CVE may have contributed to the decision not to attempt AF rhythm control [19].

Data on the effect of catheter ablation on long-term mortality and the risk of recurrent stroke increasingly indicate a protective effect [20,21]. In this context, we observed a strikingly higher rate of MACE, MACCE and all-cause death at 1 year in our cohort of AF patients with CVE. Of interest, the adverse outcome rates were significantly higher for both patients with acute and previous CVE, indicating that mortality due to acute stroke complications is not the driving mechanism. Rather, stroke-induced heart injury, for example, could play a role for long-term prognosis [22,23]. As structural remodeling of the LA was the main discriminator of patients with and without CVE in our study, further data on conditions potentially associated with systemic adverse effects such as inflammation or endothelial dysfunction would be of interest.

In contrast, the CVE group and non-CVE group did not differ significantly in terms of comorbidities that contribute to the CHA_2_DS_2_-VASc score such, as congestive heart failure, arterial hypertension, diabetes mellitus or coronary artery disease with the exception of peripheral artery disease, which was more common in the CVE group. The raw difference of 2 points in mean CHA_2_DS_2_-VASc score between CVE and non-CVE patients was mainly composed of the history of stroke. However, the CHA_2_DS_2_-VASc score has only modest predictive value for stroke (c-statistic 0.61) [7]. These findings can be ascribed to the association of AF with various non-cardioembolic stroke etiologies. In AF patients, the prevalence of carotid artery plaques has been shown to be significantly higher than in non-AF patients, ranging from 27.9% (Multi-Ethnic Study of Atherosclerosis) to 64.3% (Rotterdam Study) [24]. On the other hand, AF could be detected in 12.1% of patients with stroke attributed to large- or small-vessel disease [25]. In a multicenter case–control study in patients experiencing stroke while on treatment with non-vitamin K antagonists, 102/557 patients (22.3%) with a verified cardioembolic source of embolism had at least one competing non-cardioembolic mechanism identified [26]. Taken together, our cohort of AF patients with CVE may be a heterogeneous collective, comprising patients with stroke due to AF and ACM or other concomitant stroke etiologies.

With regard to screening on AF, it is of particular interest that, in our study, non-CVE patients had a significantly higher AF-related symptom load than patients with acute CVE. AF-related symptoms can occur either as a consequence of (tachy)arrhythmia, but also as a consequence of subsequent heart failure. In contrast, nearly all patients in the acute CVE group had no AF-related symptoms at all, as reflected by an EHRA class I. This specific finding underlines the need for a thorough search for potential silent AF in acute stroke patients, especially in those with an embolic stroke pattern. To date, there is still no consensus on how to increase the detection of silent AF in stroke patients [27,28]. A broad implementation of implantable loop recorders is limited by their invasiveness, costs and reimbursement issues, although several studies have shown the superiority of such devices in AF detection compared to conventional follow-up after stroke [29].

## 5. Limitations

This study is subject to the characteristic limitations of observational studies, such as lack of randomization or selection bias. For the group of AF patients with CVE, data on the attributed stroke etiology or imaging results confirming or ruling out an embolic stroke pattern were not available. Thus, we cannot estimate the percentage of patients with other than cardioembolic or at least concurrent stroke etiologies. As cranial imaging was not required for study participation, we have no data on potential silent brain infarcts in the non-CVE group. Patients with acute and chronic CVE were merged for primary analysis; to address potential differences in acute and chronic CVE patients, we conducted a separate subanalysis. Moreover, we cannot rule out a drop-out bias regarding the 1-year follow-up, as specific data on adverse events and AF treatment were not available for all patients. Still, the drop-out rate was similar in both groups and with 9.1% for the vital status at 1 year well within the range that would be considered ‘‘acceptable’’ for the purposes of evidence-based medicine. Follow-up data were mostly generated by phone call and relied on patients’ disclosures, although efforts to enhance the validity of information (i.e., requests for medical reports, inquiries with registration offices) were undertaken. Regarding recurrent stroke, we had no information on therapeutic adherence in patients taking OAC.

## 6. Conclusions

AF patients with acute or previous CVE are at higher risk of recurrent stroke, MACE, MACCE and all-cause death at 1 year. Assuming that AF accounted for the majority of strokes, the observation of a higher prevalence of structural remodeling in the CVE group might be a game changer concerning the need for discriminating patients at very high risk for stroke.

## Figures and Tables

**Table 1:** Patient characteristics at baseline visit.

	CVE + AF Patients	AF Patients	*p*-Value
Number of patients, n (%)	292 (14.2)	1769 (85.8)	
***Demographics***			
Age, years, mean (SD)	74.6 (9.0)	71.7 (11.5)	<0.001
Sex, male, n (%)	166/287 (57.8)	1072/1755 (61.1)	0.30
***Cerebrovascular event***		n/a	
Acute stroke at baseline, n (%)	47/291 (16.2)		
Acute TIA at baseline n (%)	11/291 (3.8)		
Previous stroke, n (%)	183/291 (62.9)		
Previous TIA, n (%)	50/291 (17.2)		
***Vascular risk factors***			
Arterial hypertension, n (%)	231/291 (79.4)	1332/1720 (77.4)	0.47
Diabetes mellitus, n (%)	86/291 (29.6)	442/1720 (25.7)	0.17
Current smoker, n (%)	14/291 (4.8)	159/1720 (9.2)	0.013
***Cardiovascular comorbidities***			
Congestive heart failure, n (%)	60/289 (20.8)	349/1682 (20.7)	1.00
CAD, n (%)	127/289 (43.9)	691/1682 (41.1)	0.36
Previous MI, n (%)	55/289 (19.0)	276/1682 (16.4)	0.27
Hypertensive cardiomyopathy, n (%)	42/289 (14.5)	313/1682 (18.6)	0.096
PAD	33/291 (11.3)	87/1720 (5.1)	<0.001
***Echocardiography***			
TTE performed, n (%)	238/292 (81.5)	1443/1769 (81.6)	0.98
LVEF, % (IQR)	55 (44; 60)	55 (45; 60)	0.055
LA diameter, mm (IQR)	46 (43; 50)	44 (40; 49)	0.001
LA diameter > 45 mm, n (%)	75/131 (57.3)	344/797 (43.2)	0.003

AF, atrial fibrillation; CAD, coronary artery disease; CVE, cerebrovascular event; IQR, interquartile range; LA, left atrium; LVEF, left ventricular ejection fraction; MI, myocardial infarction; PAD, peripheral artery disease; SD, standard deviation; TIA, transient ischemic attack; TTE, transthoracic echocardiography.

**Table 2:** AF characteristics and treatment at baseline visit.

	CVE + AF Patients	AF Patients	*p*-Value
Number of patients, n (%)	292 (14.2)	1769 (85.8)	
***AF characteristics***			
Paroxysmal AF, n (%)	172/290 (59.3)	1001/1722 (58.1)	0.71
Persistent AF, n (%)	52/290 (17.9)	424/1722 (24.6)	0.013
Long-lasting persistent AF, n (%)	66/290 (22.8)	297/1722 (17.2)	0.024
Newly diagnosed AF, n (%)	33/292 (11.3)	284/1745 (16.3)	0.03
Duration of AF, years, median, IQR §	5 (3; 9)	4 (2; 9)	0.16
AF at baseline, n (%)	125/288 (43.4)	968/1710 (56.6)	<0.001
Heart rate, median (IQR)	75 (63; 87)	78 (65; 97)	0.002
CHA_2_DS_2_-VASc score, mean (SD)	5.3 (1.5)	3.3 (1.6)	<0.001
HAS-BLED score, mean (SD)	3.0 (1.0)	2.0 (1.0)	<0.001
EHRA class			<0.001
I	153/285 (53.7)	683/1661 (41.1)	
II	104/285 (36.5)	710/1661 (42.7)	
III	24/285 (8.4)	251/1661 (15.1)	
IV	4/285 (1.4)	17/1661 (1.0)	
***AF treatment***			
Oral anticoagulation, n (%)	231/286 (80.8)	1299/1726 (75.3)	0.043
VKA, n (%)	63/286 (22.0)	355/1726 (20.6)	0.57
DOAC, n (%)	168/286 (58.7)	944/1726 (54.7)	0.20
Beta-blocker, n (%)	218/289 (75.4)	1309/1709 (76.6)	0.67
Calcium channel blocker (Verapamil-type), n (%)	36/289 (12.5)	128/1709 (7.5)	0.004
Cardiac glycoside, n (%)	32/289 (11.1)	209/1709 (12.2)	0.58
Class I antiarrhythmic drug, n (%)	5/286 (1.7)	73/1662 (4.4)	0.035
Class II antiarrhythmic drug, n (%)	13/286 (4.5)	111/1662 (7.0)	0.12
Previous cardioversion, n (%)	45/286 (15.7)	414/1705 (24.3)	0.001
Previous ablation, n (%)	25/287 (8.7)	215/1703 (12.6)	0.060
Previous PM/ICD implantation, n (%)	86/291 (29.6)	430/1741 (24.7)	0.078

§ Patients with known left atrium diameter and previous diagnosis of AF (n = 131 with CVE and n = 797 without CVE). AF, atrial fibrillation; CVE, cerebrovascular event; DOAC, direct oral anticoagulant; EHRA, European Heart Rhythm Association; ICD, implantable cardioverter defibrillator; IQR, interquartile range; PM, pacemaker; SD, standard deviation; VKA, vitamin K antagonist.

**Table 3:** Subanalysis of patients with acute versus chronic cerebrovascular event.

	aCVE + AF Patients	pCVE + AF Patients	AF Patients	*p*-Value
Number of patients, n (%)	58 (2.8)	234 (11.4)	1769 (85.8)	
***Cardiovascular comorbidities***				
Any structural heart disease, n (%)	26/58 (44.8)	196/231 (84.8)	1315/1682 (78.2)	<0.001
CAD, n (%)	14/58 (24.1)	113/231 (48.9)	691/1682 (41.1)	0.002
NYHA class I	53/57 (93.9)	68/228 (29.8)	609/1663 (36.6)	<0.001
***Echocardiography***				
LVEF, % (IQR)	55 (54; 55)	56 (49; 60)	55 (45;60)	0.051
LA diameter > 45 mm, n (%)	10/17 (58.8)	65/114 (57.0)	344/797 (43.2)	0.011
***AF characteristics***				
Paroxysmal AF, n (%)	47/58 (81.0)	125/232 (53.9)	1001/1722 (58.1)	<0.001
Persistent AF, n (%)	11/58 (19.0)	107/232 (46.1)	721 (41.2)	
EHRA class I, n (%)	53/57 (93.0)	100/228 (43.9)	683/1661 (41.1)	<0.001
***One-year event rates*** †				
1-year mortality, % (KM)	14.5 (7.5–26.9)	14.6 (10.6–20.0)	7.8 (6.6–9.2)	<0.001
MACE, % (KM)	14.6 (7.5–26.9)	15.1 (11.0–25.5)	8.1 (6.9–9.5)	<0.001
MACCE, % (KM)	18.1 (10.2–31.0)	16.0 (11.7–21.5)	8.1 (6.9–9.5)	<0.001
MACCE + major bleeding, % (KM)	18.1 (10.2–31.0)	16.4 (12.1–22.0)	8.5 (7.2–9.9)	<0.001

aCVE, acute cerebrovascular event; pCVE, previous cerebrovascular event; AF, atrial fibrillation; EHRA, European Heart Rhythm Association; LVEF, left ventricular ejection fraction; LA, left atrium; NYHA, New York Heart Association; CAD, coronary artery disease; KM, Kaplan–Meier estimator; MACCE, major adverse cardiovascular and cerebrovascular event; MACE, major adverse cardiovascular event. † Cumulative incidences at 365 days estimated by Kaplan–Meier method and compared by log-rank test.

**Table 4:** Patient characteristics at 1-year follow-up.

	CVE + AF Patients	AF Patients	*p*-Value
Number of patients, n (%)	292 (14.2)	1769 (85.8)	
***One-year event rates*** †			
1-year mortality, % (KM)	14.6 (10.9–19.4)	7.8 (6.6–9.2)	<0.001
MACE, % (KM)	15.0 (11.2–19.8)	8.1 (6.9–9.5)	<0.001
MACCE, % (KM)	16.4 (12.5–21.4)	8.1 (6.9–9.5)	<0.001
MACCE + major bleeding, % (KM)	16.8 (12.8–21.8)	8.5 (7.2–9.9)	<0.001
***Patients alive at 1-year follow-up, n***	209	1397	
***Adverse events***			
Stroke, n (%)	4/139 (2.9)	0/976 (0.0)	<0.001
TIA, n (%)	1/136 (0.7)	6/976 (0.6)	0.87
MI, n (%)	1/135 (0.7)	5/976 (0.5)	0.74
Major bleeding, n (%)	0/139 (0.0)	4/988 (0.4)	0.45
***AF treatment***			
Oral anticoagulation, n (%)	128/138 (92.8)	817/991 (82.4)	0.002
VKA, n (%)	22/137 (16.1)	146/985 (14.8)	0.7
DOAC, n (%)	105/137 (76.6)	665/985 (67.5)	0.031
Beta-blocker, n (%)	103/138 (74.6)	723/984 (73.5)	0.77
Calcium channel blocker (Verapamil-type), n (%)	27/138 (19.6)	200/984 (20.3)	0.84
Cardiac glycoside, n (%)	12/138 (8.7)	118/984 (12.0)	0.26
Class I antiarrhythmic drug, n (%)	3/136 (2.2)	35/981 (3.6)	0.41
Class II antiarrhythmic drug, n (%)	9/136 (6.6)	71/981 (7.2)	0.79
LAA occluder, n (%)	1/142 (0.7)	2/1008 (0.2)	0.27
Cardioversion, n (%)	7/142 (4.9)	85/1006 (8.4)	0.15
Ablation, n (%)	5/142 (3.5)	84/1007 (8.3)	0.044
PM implantation, n (%)	3/142 (2.1)	13/1007 (1.3)	0.43

AF, atrial fibrillation; CVE, cerebrovascular event; DOAC, direct oral anticoagulants; KM, Kaplan– Meier estimator; LAA, left atrial appendage; MACCE, major adverse cardiovascular and cerebrovascular event; MACE, major adverse cardiovascular event; MI, myocardial infarction; PM, pacemaker; TIA, transient ischemic attack; VKA, vitamin K antagonist. † cumulative incidences at 365 days estimated by Kaplan–Meier method and compared by log-rank test.

## Data Availability

The original contributions presented in the study are included in the article; further inquiries can be directed to the corresponding author.
